# Brain and lung metastasis of Bartholin’s gland adenoid cystic carcinoma: a case report

**DOI:** 10.1186/1752-1947-7-208

**Published:** 2013-08-14

**Authors:** Rajeev Ramanah, Edith Allam-Ndoul, Claire Baeza, Didier Riethmuller

**Affiliations:** 1 Obstetrics and Gynecology Department, Besancon University Medical Centre, 3 Alexander Fleming Boulevard, 25000 Besancon, France

**Keywords:** Adenoid cystic carcinoma, Bartholin’s gland, Brain metastasis, Lung metastasis

## Abstract

**Introduction:**

Adenoid cystic carcinoma of Bartholin’s gland is a very rare disease.

**Case presentation:**

A 48-year-old premenopausal woman of Caucasian origin was delivered adjuvant pelvic and inguinal radiotherapy after prior complete left Bartholin’s gland tumor excision and inguinal lymph node dissection for adenoid cystic carcinoma of Bartholin’s gland with one metastatic inguinal lymph node.

Two years after primary treatment, she presented to the Emergency Room with acute headache, hypoacousia, decrease in visual acuity, and a decrease in right leg muscle strength. A cranial magnetic resonance imaging scan demonstrated three cystic brain lesions with associated perifocal edema. Chest and abdomen computed tomography scans and a magnetic resonance imaging scan of the pelvis did not find any metastatic or residual disease elsewhere. A physical examination found no local recurrence.

Stereotactic brain biopsies with pathology examination revealed the presence of adenoid cystic carcinoma metastasis. She thus received 30Gy of brain radiotherapy but, three months later, the brain lesions did not decrease in size and left mid lobular lung lesions appeared on her chest computed tomography scan. A mid left lobe lung excision was undertaken followed by chemotherapy consisting of six cycles of cyclophosphamide, adriamycin and cisplatin. Five months after beginning chemotherapy, the brain disease progressed and our patient died.

**Conclusion:**

Our case report shows the difficulty in managing brain and lung metastasis of Bartholin’s gland adenoid cystic carcinoma as no consensus on the optimal treatment exists.

## Introduction

Bartholin’s gland carcinoma is a rare disease, accounting for 0.1% to 5% of all vulval carcinomas and representing barely 0.001% of gynecological cancers [1]. Different histological variants have been described: adenocarcinomas, squamous cell carcinomas, transitional cell carcinomas and adenoid cystic carcinomas. The latter represent 10% to 15% of Bartholin’s gland tumors and arise usually from salivary, lacrimal and nasopharynx glands, and sometimes from mammary, skin and uterine cervix glands [2]. The mean age at diagnosis is around 50 years with a range between 25 and 80 years [3]. Distant metastasis has been found in only a few cases to the lungs, liver and brain. Here, we present the first case of Bartholin’s gland adenoid cystic carcinoma (ACC) with metastasis to the brain and lung.

## Case presentation

In May 2009, a 48-year-old gravida 1 para 1 premenopausal woman of Caucasian origin presented with a tender mass in the area of the left Bartholin’s gland, associated with a left palpable inguinal lymph node, which had been slowly evolving for two years. Following the tumor’s recent increase in size and vulval pain, a left Bartholin’s gland excision (bartholinectomy) was performed under general anesthesia.

The pathology examination revealed a primitive ACC of Bartholin’s gland measuring 30mm but with positive margins.

Upon diagnosis of this unexpected malignant lesion, a systematic search for metastatic disease was undertaken. Chest, abdomen and pelvis computed tomography (CT) scans with intravenous contrast along with a magnetic resonance imaging (MRI) scan of the pelvis showed a highly suspicious left inguinal lymph node demonstrating high metabolic activity upon whole body positron emission tomography (PET) scan. No other site of metastatic disease was detected.

Next, our patient underwent radical tumor re-excision with left inguinofemoral lymph node dissection. The postoperative pathology examination found two remaining tumors of 6mm close to the surgical margin. Tumor cavity vaginal and vestibular re-excisions were clearly disease-free. Among the seven inguinofemoral lymph nodes collected, one of them, measuring 45mm with an intact capsule, had metastatic disease.

Postoperatively, after multidisciplinary discussion of the case, adjuvant pelvic and inguinal radiotherapy was recommended. She thus obtained 66Gy of radiation.

Follow-up visits were organized every three months with CT scans of the chest, abdomen and pelvis performed every six months. In June 2011, two years after her primary treatment, she presented to the Emergency Room with acute headache, hypoacousia, decrease in visual acuity, and a decrease in right leg muscle strength.

**Figure 1:**
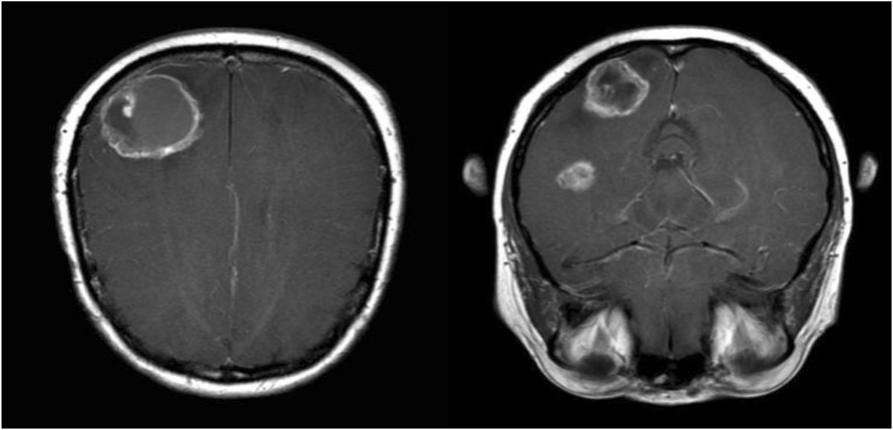
Gadolinium-enhanced axial T1-weighted brain magnetic resonance imaging scans showing three metastatic cystic brain lesions in the right parieto-occipital and temporal regions with associated perifocal edema.

A cranial MRI scan demonstrated three cystic brain lesions in the right parietal, left parieto-occipital and left temporal region with associated perifocal edema (Figure 1). Chest and abdomen CT scans and a MRI scan of the pelvis did not find any metastatic or residual disease elsewhere. A physical examination found no local recurrence.

**Figure 2:**
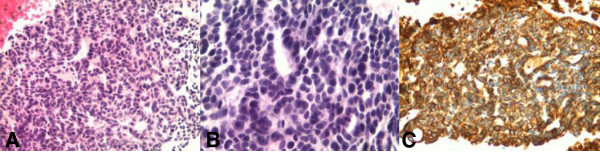
**Histology of the brain metastasis biopsy specimen. (A)** Hematoxylin and eosine stain with magnification x 200: adenoid cystic carcinoma cells (purple) adjacent to normal brain tissue (pink) at the upper left. **(B)** Hematoxylin and eosine stain with magnification x 400: adenoid cystic carcinoma cells (purple) arranged in a typical cribriform pattern, forming columns around gland-like spaces. **(C)** Immunohistochemistry with anti-cytokeratin 7 antibodies showing a typical positive stain for adenoid cystic carcinoma cells.

Stereotactic brain biopsies were undertaken. A pathology examination revealed the presence of ACC metastasis with characteristic tumor proliferation in a cribriform pattern composed of nests and columns of cells arranged concentrically around gland-like spaces filled with eosinophilic periodic acid-Schiff-positive material. These results were confirmed after immunological staining with specific antibodies, which showed that the brain lesions had similar characteristics as the initial ACC of Bartholin’s gland that was diagnosed two years earlier (Figure 2). Immunohistochemical characteristics were: cytokeratin (CK) AE1/AE3 positive, CK7 positive, epithelial membrane antigen (EMA) positive, S100 weakly positive, CK20 negative, estrogen receptor negative, progesterone receptor weakly positive.

**Figure 3:**
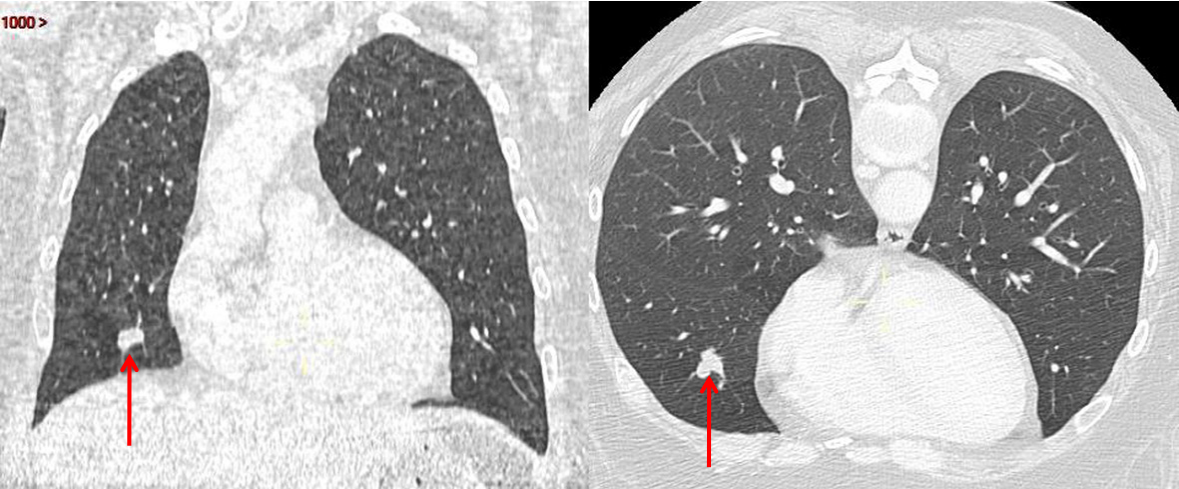
Frontal and axial chest computed tomography scans showing adenoid cystic carcinoma metastasis (red arrow) in the right middle lobe of the lung.

Our patient’s case was once again discussed at a multidisciplinary meeting where whole brain radiotherapy was advocated. The patient thus received 30Gy of brain radiotherapy ending in August 2011. A cranial MRI scan, chest and abdomen CT scans, and a MRI scan of the pelvis were performed three months later to evaluate the efficacy of the treatment. The brain lesions had not decreased in size and right middle lobe lung lesions appeared on the chest CT scan (Figure 3). A whole body PET scan, pelvic imaging and examination did not show any local recurrence or other metastatic lesions.

A lung biopsy with an extemporaneous examination followed by a middle right pulmonary lobe excision was undertaken. A postoperative pathologic examination confirmed the diagnosis of ACC lung metastasis. Afterward, our patient received chemotherapy consisting of cyclophosphamide, cisplatin and adriamycin. Five months after beginning chemotherapy, the brain disease progressed with the appearance of frequent seizures upon increased cerebral edema, and our patient died.

## Discussion

An ACC of the Bartholin’s gland presents itself with similar symptoms as most vulval cancers. Patients usually complain of pruritus, a burning sensation and pain, which are explained by this slow-growing tumor’s tendency for local and perineural invasion. These symptoms can even be experienced before the physical appearance of any vulval mass. Among other nonspecific signs like bleeding, dyspareunia, and/or discharge from an abscess [4], the presence of an inguinal lymph node should be considered as being highly suspicious of malignancy. However, in most cases of symptomatic Bartholin’s cysts, patients will be treated with only drainage and marsupialization as the diagnosis of a benign condition is expected. That is why some authors recommended performing fine-needle aspiration cytology before or during surgery, especially in women over the age of 40 [5]. However, the diagnostic value of cytology is poor. Moreover, the overall incidence of Bartholin gland carcinoma has been found to be low and similar in pre- and postmenopausal women [6]. Advocating systematic excision of a Bartholin’s cyst would probably create excess morbidity and complications associated with radical procedures while the primary surgery would still result in inadequate margin status. A reasonable approach to the management of any patient with Bartholin gland mass may be to incise, drain and obtain multiple biopsies to rule out any underlying carcinoma. In our case report, a reintervention was necessary for positive margins and inguinofemoral lymphadenectomy although a complete Bartholin gland excision was performed as the primary treatment. Radical vulvectomies should not be systematically warranted because recurrence rates are similar in patients with positive and negative margins, explained in part by adjuvant radiotherapy being preferentially proposed in case of inadequate margin status [4]. Instead, surgery and radiotherapy should be tailored to the extent and location of the tumor so as to decrease morbidity and to maintain an acceptable quality of life without compromising the survival rate, as reported in a survey over 20 years [7].

This case report, with metastatic brain and lung diseases, especially without any local recurrence, does not correspond to the usual presentation of ACC, except for the age at diagnosis. In a review of the literature published by Alsan *et al.*[8], the lungs were found to be the most common site of distant metastasis, followed by the liver and less frequently the bone. Until now, only one case of brain metastasis has been described in the English literature [9]. ACC is known to have high rates of local recurrence estimated at 30% and with a risk of distant metastasis being at 31% [8]. Ten-year disease-free interval and survival rates are respectively 38% and 50% [4]. Here, no local recurrence was observed, possibly because our patient had an optimal local surgical and radiation treatment, but brain metastasis occurred two years after primary treatment.

Brain metastases from gynecological cancers are rare, and usually occur in cases of widely disseminated carcinomas through the bloodstream or the lymphatic system. In our case, it is most probably through the lymphatic system that tumor cells metastasized to the brain. In the previous case report of Bartholin’s gland, ACC brain metastasis was described as a single lesion in the cerebellar vermis [9]. The authors were thus able to surgically remove the lesion before whole brain radiotherapy. However, postoperatively, the patient experienced tetraplegia and an MRI scan revealed residual tumors in the vermis, the cerebellar and cerebral cortices. These residual brain lesions were decreased with radiotherapy. It was not clear whether surgery was responsible for the seeding metastases or new cortical lesions developed postoperatively.

Hatiboglu *et al.* demonstrated that brain metastasis from an ACC of the Bartholin gland responded well to radiotherapy at a total dose of 30Gy but the follow-up interval was only one month [9]. We could not find the same efficacy, probably because of the presence of multiple lesions that could not be surgically removed. Indeed, a randomized controlled trial showed that patients with a single brain metastasis treated with surgery and radiotherapy had a longer survival and a lower recurrence rate than patients treated with radiotherapy alone [10]. However, in cases of multiple or inoperable brain metastases, whole brain radiotherapy is recommended [10]. In the present case, there were three lesions in the cerebral cortex making surgery difficult and risky. Thus radiotherapy as a primary treatment proved inadequate since it had no effect on the brain lesions and lung metastasis appeared. After the middle pulmonary lobe resection, chemotherapy helped control disease progression in the lungs but the disease progressed in the brain. Previous management of lung metastasis showed that surgery alone was not sufficient [4]. Several chemotherapeutic regimens have been reported in the literature but the use of cyclophosphamide, adriamycin and cisplatin seems most common [4,11].

## Conclusions

The prognosis of ACC with brain and lung metastasis is poor. Although, local and distant recurrences of ACC have been reported to be radiosensitive [12], we did not achieve any disease control. It is actually not possible to have large cohort or randomized studies on the ACC of Bartholin’s gland because of its low prevalence, making it difficult to obtain a consensus regarding its optimal treatment. This case study showed that in cases of multiple brain metastases, radiotherapy alone is probably not sufficient, thus raising the question of an early association of chemotherapy.

## Consent

Our patient died and next-of-kin was not traceable after the authors undertook every effort to trace the family following the conditions specified by the Committee on Publication Ethics’ (COPE) code of conduct. Every effort was made to protect the identity of our patient and there is no reason to believe that our patient would have objected to publication.

## Abbreviations

ACC: Adenoid cystic carcinoma; CK: Cytokeratin; CT: Computed tomography; EMA: Epithelial membrane antigen; MRI: Magnetic resonance imaging; PET: Positron emission tomography.

## Competing interests

The authors declare that they have no competing interests.

## Authors’ contributions

RR analyzed data and wrote the manuscript. EAN and CB performed the literature search. DR reviewed the manuscript. All authors read and approved the final manuscript.

## Authors’ information

RR is a gynecologist specialized in vulval cancer treatment and research. He is a fellow of the International Society for the Study of Vulvovaginal Disease (ISSVD).
